# Opening the operating theatre to oncology: treatment of locally recurrent rectal cancer with peritoneal metastasis by simultaneous hyperthermic intraperitoneal chemotherapy and intraoperative electron beam radiotherapy

**DOI:** 10.1308/rcsann.2024.0111

**Published:** 2025-06-11

**Authors:** Y Salem, CT West, M West, S Jain, H Yano, J Smith, C Green, A Bateman, A Mirnezami

**Affiliations:** ^1^ University Hospital Southampton NHS Foundation Trust, UK; ^2^ University of Southampton, UK; ^3^ NIHR Southampton Biomedical Research Centre, Perioperative Medicine and Critical Care Theme, University Hospital Southampton, UK

**Keywords:** Locally recurrent rectal cancer, Peritoneal metastasis, IOERT, CRS–HIPEC, Total pelvic exenteration

## Abstract

Despite advances in multimodality therapies and surgical technique, locally recurrent rectal cancers (LRRC) and those with peritoneal metastasis (PM) remain challenging for surgeons and oncologists. Although total pelvic exenteration (TPE) and cytoreductive surgery (CRS) for peritoneal disease may enable removal of the main tumour mass and macroscopically visible peritoneal spread, respectively, local recurrence from an R1 resection, and peritoneal recurrence from microscopic peritoneal disease, remain key concerns. Combining radical surgery with oncological adjuncts such as intraoperative electron beam radiotherapy (IOERT), and hyperthermic intraperitoneal chemotherapy (HIPEC), may improve outcomes in this field. Nevertheless the application of both together is poorly reported. Here we describe a case of LRRC with PM treated with TPE and simultaneous HIPEC and IOERT.

## Background

Colorectal cancer is a common health problem with an increasing global burden, and is the second leading cause of cancer-related deaths.^1^ Locally advanced rectal cancer extending beyond the mesorectum or locally recurrent rectal cancer (LRRC), combined with peritoneal metastasis (PM), has been considered a sign of poor prognosis and perhaps incurable.^2^ However, advances in multimodality treatments, contemporary neoadjuvant and adjuvant chemotherapies and radiotherapies, and radical exenterative pelvic surgery (TPE), have each led to incremental improvements in oncological outcomes and survival in carefully selected patients.^3,4^

**Figure 1:**
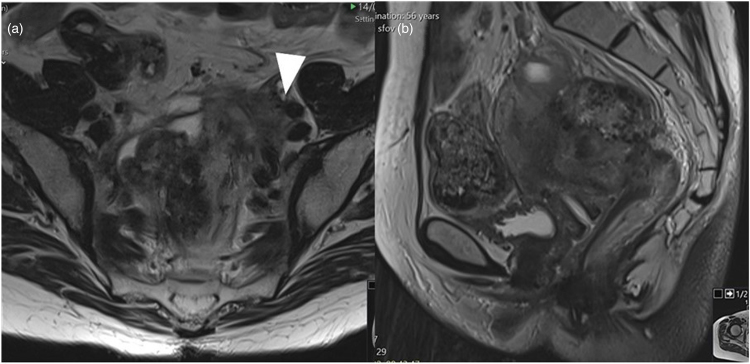
Axial (a) and sagittal (b) pelvic magnetic resonance imaging showing locally recurrent rectal tumour with disease inseparable from the external iliac vessels (arrow head).

**Figure 2:**
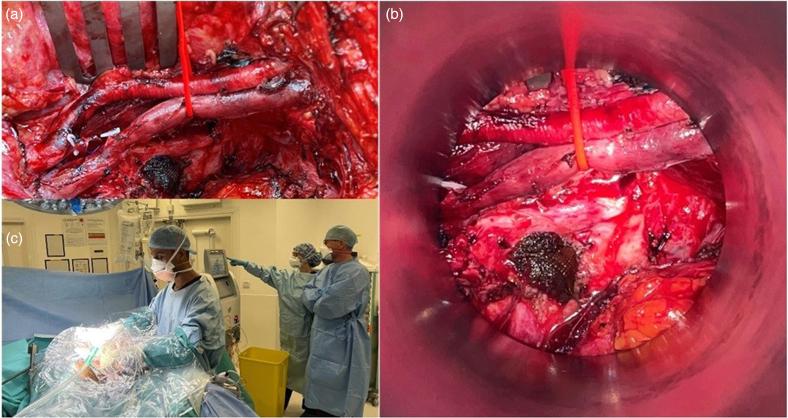
(a) Operative view taken after tumour resection showing the left pelvic sidewall with preservation of the non-expendable external iliac vessels but with resection of the internal iliacs and branches. (b) Operative view of the same field after deploying the intraoperative electron beam radiotherapy (IOERT) applicator and showing the site of IOERT treatment. (c) Following total pelvic exenteration, cytoreductive surgery and IOERT, hyperthermic intraperitoneal chemotherapy is delivered via the open coliseum technique.

PM in particular is regarded as a poor prognostic feature and is detected synchronously in 5% of patients undergoing curative surgical resection of colorectal cancer, and metachronously in 6%.^5^ Historically, treatment has centred on systemic chemotherapy in addition to palliative surgery on a case-by-case basis; however, this has yielded poor outcomes with a median survival of 5–7 months only. The introduction of cytoreductive surgery and hyperthermic intraperitoneal chemotherapy (CRS–HIPEC) has led to improved oncological outcomes in this field.^3^ Another poor prognosis feature is involvement of the pelvic sidewall structures by LRRC and this has been found to greatly increase the chances of a positive cancer resection margin (R1).^4,6,7^ In such circumstances, intraoperative electron beam radiotherapy (IOERT), which exposes the most at-risk anatomical area for an R1 resection to direct radiation exposure, may reduce the risk of local recurrence.^4,8^ Combining radical surgery with oncological adjuncts such as IOERT, and HIPEC, may improve outcomes in this field; however, the simultaneous application of both together is poorly reported.

In this report, we describe a case of LRRC that had progressed following neoadjuvant treatment involving the pelvic sidewall and with peritoneal metastases, and was the first case to be treated successfully with TPE surgery combining CRS–HIPEC and IOERT in the United Kingdom.

## Case history

A 58-year-old woman underwent an emergency Hartmann’s procedure for an acute perforated rectosigmoid adenocarcinoma. Histopathologically this was found to be a T4a N0 M0 R0 MMR-proficient, KRAS mutant and BRAF wild-type adenocarcinoma. Subsequently, the patient initially received adjuvant chemotherapy with capecitabine, switched to raltitrexed because of cardiotoxicity. She subsequently entered a surveillance programme. However, within 6 months, endoscopy and imaging showed evidence of biopsy-confirmed local and peritoneal recurrence. Computed tomography (CT) of the chest, abdomen and pelvis, magnetic resonance imaging (MRI; Figure 1) of the pelvis and positron emission tomography (PET) showed a complex multifocal pelvic recurrence as well as peritoneal metastases. The pelvic disease involved a main soft tissue mass in the Pouch of Douglas contiguous with the top of the rectal stump and involving the uterus and extending into the left pelvic sidewall and presacral fascia, with bilateral PET-avid pelvic sidewall nodes. Close contact with external iliac vessels was observed. Suspected multifocal peritoneal disease was noted on the PET imaging, which was suggestive of low-volume metastases.

The patient was discussed at a regional complex cancer multidisciplinary team meeting, and diagnostic laparoscopy, peritoneal cancer index (PCI) assessment and subsequent neoadjuvant therapy were recommended.

Diagnostic laparoscopy and PCI assessment revealed a low PCI (score = 6; low 1–10, intermediate 11–20, high 21–39). The patient completed neoadjuvant chemoradiotherapy, receiving 50.4Gy in 28 daily 1.8Gy fractions with concurrent chemotherapy. Subsequently, CT and MRI re-staging were performed, which unfortunately revealed further progression of the disease. An increase in tumour size causing left ureteric obstruction, further extension into the left pelvic sidewall and an increase in the size of the peritoneal deposits were noted.

The patient was discussed again and was still deemed suitable for radical surgery, including HIPEC and IOERT, to reduce the risk of both peritoneal and local recurrence, respectively.

During surgery, the right colon and terminal ileum were also found to be involved. The patient underwent a total infralevator pelvic exenteration, with en bloc left pelvic sidewall resection, taking the internal iliac vessels at the origin while preserving the common and external iliac vessels. A partial right pelvic sidewall resection, right parietal peritonectomy, en bloc right hemicolectomy and excision of the two further peritoneal deposits was performed.

IOERT (10Gy boost in one fraction to the left pelvic side wall with a 9MeV/no bolus/9cm cone size/ 30 degrees bevelled angle applicator protocol to a 2cm depth), and HIPEC with mitomycin C heated to 42°C were also conducted (Figure 2). CC0 resection of the peritoneal disease was achieved.

The final histopathological findings revealed recurrent moderately differentiated rectal adenocarcinoma with evidence of partial tumour regression extending into the pelvic sidewall. There were peritoneal metastases and the pelvic sidewall nodes were negative (N0), although signs of regression were noted in some of these nodes. The closest margin on the recurrent tumour excision was the posterolateral margin at the site of the non-expandable vessels, with a 0.9mm margin (R1) resection.

Her postoperative course was uneventful, except for a prolonged postoperative ileus, and no Clavien–Dindo grade 3 or higher complications were noted. The patient was enrolled in a surveillance programme as per local protocols. To date, no recurrence has been detected at three years following surgery.

## Discussion

Achieving an optimal surgical oncology goal in LRRC with evidence of disease progression following neoadjuvant chemoradiotherapy, involving the pelvic sidewall and in the presence of peritoneal metastases, is challenging. However, with the extension of multimodality care into the operating theatre, providing radical surgical oncology combined with IOERT and HIPEC, several limitations of conventional care were mitigated, which may help improve outcomes.

Disease progression following neoadjuvant chemoradiotherapy has long been established as a particularly poor prognostic indicator and may take place in up to 15% of patients. These patients have been noted to have higher rates of both local and systemic relapse.^9^ In the present case, progression was noted both at the site of the local recurrence as well as in the peritoneal metastases.

Extension of LRRC into the pelvic sidewall is a further poor prognostic variable. Moore *et al* and others have long noted that this confers a greater risk of a positive cancer resection margin, and translates to poorer survival outcomes.^6,7,10^ In more recent times, en bloc resection of the pelvic sidewall has been increasingly described, resecting neurovascular structures such as the internal iliac vasculature and or sciatic nerve roots, in an effort to enhance R0.^10^ In the present case, an anticipated positive resection margin (R1) was obtained on the non-expendable external iliac vessels; however, resection and reconstruction of these in the presence of peritoneal disease was felt to not be fitting given the potential morbidity. As a result, IOERT was utilised to mitigate for this R1. IOERT represents a specialised form of focused radiotherapy that permits treatment intensification to the cancer bed and other areas at risk of a positive resection margin, while limiting the deleterious effects of radiotherapy on normal tissue by shielding it from the treatment beam. Although IOERT may be associated with anastomotic and wound complications, systematic reviews and meta-analyses suggest that it may improve oncological outcomes in cases of R1 resection.^4,8^

A further poor prognosis feature of the present case was the presence of peritoneal metastases. This has traditionally conferred a dismal prognosis, and conventional systemic chemotherapy combined with palliative surgery has had poor survival outcomes, with median overall survival times of 5–7 months only.^3^ Since the 1990s, however, pioneering approaches have sought to employ novel strategies for this condition. Cytoreductive surgery, popularised by Sugarbaker, is now applied for selective cases offered at specialist units and aims to remove all macroscopically visible peritoneal spread through peritonectomy or visceral resections, whereas HIPEC is advocated to eradicate microscopic PM not removed as part of a CRS.^11^ The combination of CRS–HIPEC is currently regarded as the standard of care for such cases of medium to low PCI disease.^12^

Until now there has been only one other publication internationally on the simultaneous combination of IOERT and HIPEC in the setting of LRRC, with 16 cases being described in a single-centre cohort study from a Dutch tertiary referral centre.^13^ No mortality was noted in this seminal series; however, as expected from the invasiveness of IOERT and HIPEC combined with TPE, severe postoperative complications were noted in 57% of patients, with infected fluid collections representing the most commonly observed complication. This study concluded that the combination of CRS–HIPEC and IOERT was safe and feasible in selected patients and could lead to long-term survival.

## Conclusions

In patients with LRRC with high-risk features for R1 resection and synchronous PM, the continuation of multimodality care into the operating theatre, combining radical exenterative surgery, IOERT and HIPEC, may offer the advantages of conventional multimodality care. This concept of opening the operating theatre to oncology represents a natural extension of the established principles of multimodal and multidisciplinary care, and allows further treatment escalation in the most challenging of cases. However, larger and better quality studies are required to identify the patients most suitable for this approach, and to better determine the magnitude of any incremental gains afforded.

## Data Availability

The original contributions presented in the study are included in the article or supplementary material. Further enquiries can be directed to the corresponding author.
